# Time-of-Flight Secondary Ion Mass Spectrometry Coupled with Unsupervised Methods for Advanced Saffron Authenticity Screening

**DOI:** 10.3390/foods13132033

**Published:** 2024-06-27

**Authors:** Elisabetta De Angelis, Omar Al-Ayoubi, Rosa Pilolli, Linda Monaci, Alice Bejjani

**Affiliations:** 1 Institute of Sciences of Food Production, National Research Council of Italy, Via G. Amendola 126/O, 70126 Bari, Italy; elisabetta.deangelis@ispa.cnr.it (E.D.A.); rosa.pilolli@ispa.cnr.it (R.P.); 2 Lebanese Atomic Energy Commission, National Council for Scientific Research, Riad El Solh, Beirut 107 2260, Lebanon

**Keywords:** saffron, safflower, turmeric, authenticity, adulteration, TOF-SIMS, mass spectrometry, PCA, spices, multivariate analysis

## Abstract

Saffron, renowned for its aroma and flavor, is susceptible to adulteration due to its high value and demand. Current detection methods, including ISO standards, often fail to identify specific adulterants such as safflower or turmeric up to 20% (*w*/*w*). Therefore, the quest continues for robust screening methods using advanced techniques to tackle this persistent challenge of safeguarding saffron quality and authenticity. Advanced techniques such as time-of-flight secondary ion mass spectrometry (TOF-SIMS), with its molecular specificity and high sensitivity, offer promising solutions. Samples of pure saffron and saffron adulterated with safflower and turmeric at three inclusion levels (5%, 10%, and 20%) were analyzed without prior treatment. Spectral analysis revealed distinct signatures for pure saffron, safflower, and turmeric. Through principal component analysis (PCA), TOF-SIMS effectively discriminated between pure saffron and saffron adulterated with turmeric and safflower at different inclusion levels. The variation between the groups is attributed to the characteristic peaks of safflower and the amino group peaks and mineral peaks of saffron. Additionally, a study was conducted to demonstrate that semi-quantification of the level of safflower inclusion can be achieved from the normalized values of its characteristic peaks in the saffron matrix.

## 1. Introduction

Spices are natural plant products containing pungent or aromatic substances and are widely used as a food flavor enhancer. Their trade has one of the longest and richest histories of any industry. Among them, saffron represents one of the most valuable and highly appreciated spices throughout the world mainly due to its peculiar aroma, flavor, taste, and color. Saffron derives from the flower of *Crocus sativus*, which consists of three red stigmas that are consequently collected and dried under special conditions to produce the final spice. Saffron is cultivated in many environments characterized by different pedo-climatic conditions such as Iran, India, Afghanistan, Greece, Morocco, Spain, and Italy, with Iran representing the largest producer in the world, covering 90% of the global production. Its global production is estimated at 418 t y^−1^ [1]. According to the producer state, specific agronomical practices (corm planting, fertilization, and irrigation) and post-harvest techniques (drying and storing) are adopted for this spice production, leading to a number of types of saffron with unique characteristics and quality traits. In addition to being an excellent culinary spice, saffron is renowned for its constituent’s biological activity and health-promoting properties, including anticonvulsant [2], anti-inflammatory [3], antitumor [4], anti-oxidant [5], antiatherogenic [6], and antidepressant [7] activity, as well as enhancing learning and memory capacity [8,9]. Major compounds contributing to the color, taste, and aroma of saffron are crocin, picrocin, and safranal. Specifically, crocin (C_44_H_64_O_24_, water-soluble crocetin esters) is responsible for the strong coloring capacity, picrocrocin (C_10_H_14_O_7_, monoterpene glycoside, precursor of safranal) imparts the bitter flavor, and safranal (C_10_H_14_O) provides the characteristic odor and aroma [10]. Over 150 constituents have been reported to chemically characterize the stigma of saffron, including lipophilic carbohydrates; proteins; minerals; mucilage; starch; gums; vitamins; alkaloids; xanthone carotenoid; mangicrocin; saponins; and many pigments such as α and β carotenes [4]. Saffron quality strictly depends on several factors, including agronomical growing conditions; post-harvest techniques; and crocin, picrocrocin, and safranal contents. According to ISO (3632 1, 2:2010) [11], saffron quality is classified into three categories, namely I (high quality), II (medium quality), and III (low quality), based on specific ranges of the three molecules crocin, picrocrocin, and safranal. In general, the higher the content of these three main compounds is, the higher the quality of saffron. Due to the high price and labor required for plantation and production, along with the increasing global demand for this spice, cases of saffron adulteration have dramatically increased in recent years [12]. Saffron can be adulterated in different ways, including the mixing with natural or biological compounds such as flour, species of other plants (*Calendula officinalis*, *Carthamus tinctorius* L., *Gardenia jasminoides Ellis*, etc.), powdered spices of plant origin (*Curcuma longa*, etc.), animal origin compounds (shredded and dyed meat fibers or dipping with honey), chemical-based or synthetic compounds such as artificial dyes (carminic acid, Ponceau 4R, Sudan (I-IV), etc.), or saffron floral parts (style, stamen of wild ancestral or variant of saffron) [12].

As already reported, the ISO 3632-2 protocol, issued by the International Organization for Standardization, is the current standard method for saffron quality analysis [11]. This method combines UV–vis spectroscopy and high-performance liquid chromatography (HPLC) to determine color strength and crocin content, ultimately defining the saffron quality level. Despite its widespread use, it has been demonstrated that saffron adulterants (safflower, marigold, or turmeric) were not detected by the ISO normative by up to 20% (*w*/*w*) [13]. Numerous efforts have been made by researchers to develop robust screening methods for identifying saffron adulteration. To date, several analytical methodologies relying on different approaches have been developed to detect saffron fraud, including physical methods, chromatographic techniques, spectroscopic techniques, molecular or DNA-based methods, and sensor-based techniques, as recently reviewed by Kumari et al. (2021) [12]. Although accurate and readily available, each technique may suffer from limitations related to the nature of the adulterants, sample loss, time-consuming sample preparation, and concentration level of adulterants. Moreover, they are not suitable for online quality analysis, especially in large-scale and industrial applications [12,14,15].

A metabolomics-integrated approach serves as an alternative strategy for detecting saffron adulteration, employing untargeted methods for comparative studies and classification through multivariate statistical analysis. Techniques such as untargeted analysis, ambient mass ionization, high-resolution mass spectrometry (HRMS), and non-targeted NMR-based metabolomics are explored to identify potential markers for authenticity assessment and the detection of emerging frauds [16,17,18,19,20,21]. In recent work, we applied, for the first time, direct analysis in real-time (DART) ionization coupled with an untargeted HRMS approach using an Orbitrap mass analyzer to assess saffron authenticity [22]. Our method effectively discriminated between pure saffron and adulterated samples even at low inclusion levels (5–10%), which would not be traceable with the official ISO normative.

Continuing our exploration of advanced techniques for assessing saffron authenticity, we expanded our analytical methods to include the time-of-flight secondary ion mass spectrometry (TOF-SIMS) technique. This technique provides a relatively high mass spectral signature of the outer surface while offering lateral chemical distribution with minimal prior preparation. It is renowned for its molecular specificity and high sensitivity [23,24,25]. Additionally, it is considered a non-destructive technique that analyzes samples on a microscale [26]. Compared to conventional techniques, TOF-SIMS is particularly valuable when samples are scarce and costly. Moreover, it is environmentally friendly, requiring no extraction or complicated chemical sample preparation.

The application of this technique in food analysis has undergone significant development over the last two decades. The distribution of capsaicin, the heat-triggering compound, in Capsicum peppers was investigated using the TOF-SIMS technique in imaging mode [27]. It was found to be concentrated in the pockets between the outer part of palisade cells and the septum cuticle, as well as in the intercellular spaces of the placenta and the intraocular septum. TOF-SIMS has also been used to detect chlorinated pesticides on the surface of cultivated mushrooms and in olives [28,29]. Through multivariate analysis, particularly PCA, TOF-SIMS effectively discriminated between untreated olives with pesticides and those that had been treated, whether washed or unwashed before analysis. In another application using TOF-SIMS and PCA, researchers demonstrated that the extraction method for the color bixin from Orellana fruit seeds at 130 °C is not the most effective [30]. Their analysis, incorporating sample heating, revealed that the degradation of this component begins at 70 °C. Through PCA, they demonstrated structural changes in the bixin molecule with varying heating temperatures. Piras et al. [31] examined the chemical composition of Sardinian myrtle’s alcoholic extract, and PCA distinguished two groups based on the concentrations of triacontanoic acid and other compounds. 

We applied the TOF-SIMS technique coupled with principal component analysis to discriminate between pure saffron and adulterated saffron with safflower and/or turmeric at concentration levels below 20%. Our objective was to establish a fast, simple, and robust screening analytical methodology for detecting plant adulterants in saffron samples. Therefore, in this study, we investigated and developed a simple and straightforward protocol based on directly analyzing a minimum portion of the solid sample by skipping any prior treatment, aiming to achieve robust results in a short time. For statistical analysis, data mining was carried out by using the multivariate data analysis software specifically designed for TOF-SIMS data. 

To our knowledge, saffron, turmeric, and safflower powders have not been previously analyzed using TOF-SIMS. Hence, the signatures of the dried stigma of *Crocus sativus* L. (saffron), the dried petals of *Carthamus tinctorius* plant (safflower), and the dried rhizome of *Curcuma longa* plant (turmeric) are presented for the first time. Although TOF-SIMS is recognized more for its qualitative capabilities than its quantitative precision, primarily due to the matrix effect [32], our exploration was extended to a semi-quantitative analysis of safflower in the saffron matrix. The study and discussion of the semi-quantification of safflower in the saffron matrix provide novel insights into the fast identification of this potential adulterant.

## 2. Materials and Methods

### 2.1. Sample Preparation

Pure Italian saffron powdered samples (n = 6) derived from different regions of origin and adulterated samples were obtained from the Food Integrity consortium. Pure and adulterated samples with safflower or turmeric at different inclusion levels, namely 5%, 10%, and 20% (n = 3 for each level), were analyzed without any prior treatment. Pure safflower and turmeric powder, certified by a Lebanese supplier from India, were also purchased and analyzed in parallel. Samples were stored at room temperature in the dark before use. Less than 15 mg of powder from each reference was deposited and lightly pressed using a spatula onto a double-sided adhesive tape. The other side of the tape was affixed to an aluminum support, which was secured with clamps onto a back-mount sample holder capable of accommodating 16 samples. A maximum of six samples from the same set were loaded for each run.

### 2.2. TOF-SIMS Analysis

Spectral analysis in this study was performed using a TOF-SIMS V instrument (IONTOF GmbH, Münster, Germany). The samples were analyzed using a 25 keV liquid metal ion gun source. A flood gun was used to provide low-energy electrons (20 eV) for compensating any surface charge. The powder samples were raster scanned randomly with an analyzed area of 500 × 500 µm^2^ in three to six different areas. A 0.1 pA Bi_3_^+^ beam was used for 1000 s, maintaining a total primary ion beam dose below 5 × 10^11^ ions/cm^2^ to ensure static conditions [32]. Throughout the analysis, beam intensity was monitored before and after each session, with continuous monitoring of peak emission to ensure no more than a 5% loss occurred. Data acquisition in positive and negative polarity modes and subsequent processing were performed using SurfaceLab 6.7 (ION-TOF GmbH, Münster, Germany). Mass calibration was carried out using H^+^, C_2_H_3_^+^, C_2_H_5_^+^, ^41^K^+^, C_2_H_5_O^+^ peaks for positive mode and using CH^−^, OH^−^, C_2_^−^, C_4_H^−^ for negative mode. All recorded spectra were offline corrected using the advanced TOF correction feature to gain mass resolution. Typical mass calibration was between 2000 and 3500 on C_3_H_5_^+^ ion and between 2400 and 3800 on C_4_H^−^. This is considered a reasonable mass resolution for insulating samples having irregular surfaces and void spaces between the powder particles. The peak assignment was based on the available literature and choosing the compound with the lowest mass deviation in ppm among the proposed assignments by the software library.

### 2.3. Statistical Analysis

A multivariate statistical approach was used for data processing in order to improve the separation between the different sample groups. Principle component analysis (PCA) was accomplished using Spectragui, an external software from NESCA/Bio, Washington University, that is adapted for IONTOF data [33]. PCA is a robust and versatile multivariate statistical method capable of reducing dimensionality and revealing patterns. It transforms a dataset of correlated variables into a new set of uncorrelated (orthogonal) variables known as principal components (PCs). This transformation results in a reduction in variables, as the new PC axes are defined through the recombination of the original variables. The significance of each PC is determined by the amount of variance it captures. The results of the analysis are presented through score and loading plots. The former indicates the projection of a sample onto PCs, while the latter represents the projection of original variables onto the new PCs. Consequently, the loading plot provides the contributions of the original variables to the newly created PCs, elucidating which variables drive the observed differences within the samples. Furthermore, the score plot of two PCs provides insights into the samples’ positioning in the multivariate space, showing the 95% confidence limit for each group.

## 3. Results and Discussion

### 3.1. Characterization of Pure Saffron, Safflower, and Turmeric 

**Figure 1:**
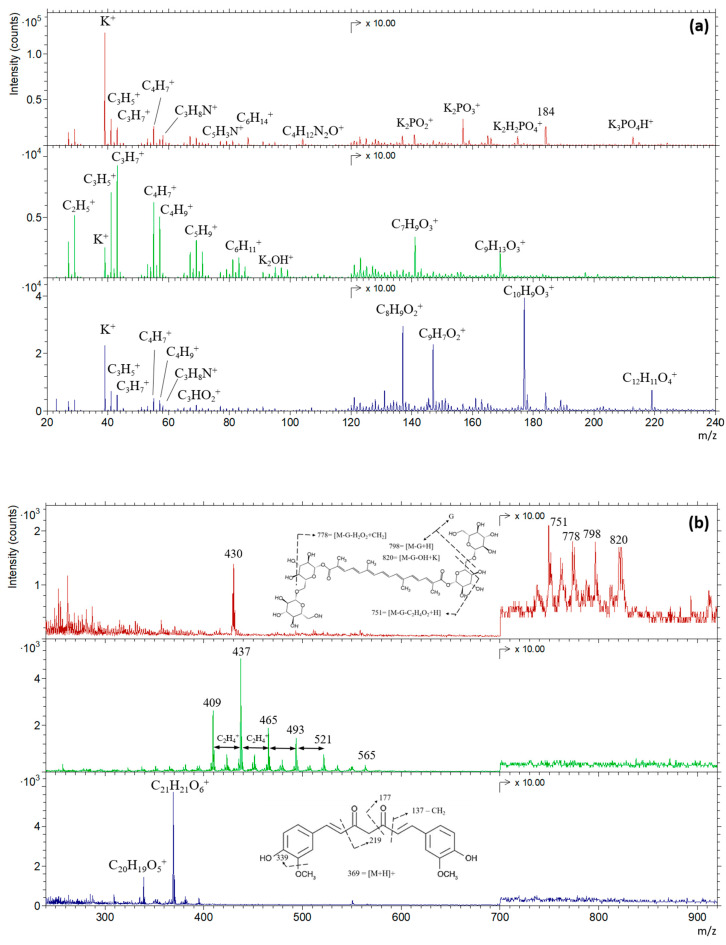
Positive TOF-SIMS spectra of pure saffron (red), safflower (green), and turmeric (blue) are shown in regions (**a**) 20–240 *m*/*z* and (**b**) 240–940 *m*/*z* with individual intensity scales. For visual clarity, the regions above 120 u.m.a and above 700 u.m.a are multiplied by 10 consecutively. The chemical structures of the crocin1 molecule, as well as the curcumin molecule and the putative fragmentations, are presented in the red and blue spectra, respectively.

The positive ion spectra of pure saffron, safflower, and turmeric powders are offset overlaid in Figure 1a,b. For the PCA, only the positive spectra were utilized and, therefore, are presented and discussed in this work.

As these samples were analyzed for the first time using the TOF-SIMS technique, individual negative and positive spectra of each reference are provided in the Supplementary Information (Figures S1–S3). Although peak identification is not the main focus of this study, mass assignments were tentatively carried out based on mass resolution and by combining information from spectra acquired in positive and negative ion modes, along with referencing the available literature for some components. It is worth noting that, in the literature, the interest in saffron, safflower, and turmeric varies significantly in terms of purpose, type of analysis, and the specific part of the plant under study. For instance, research on safflower predominantly focuses on its seeds rather than other parts, including the flower. Additionally, most studies mostly investigate the bioactive compounds within their respective botanical families, pushing the boundaries of research by reporting new discoveries and identification methods of new compounds belonging to these categories [34,35,36,37,38,39,40,41,42]. Table S1 consolidates information from various types of studies, such as proximate and phytochemical analyses, highlighting the most prevalent compounds or families of compounds for each product, along with their potential concentrations. In Table S1, all chemical compounds useful for our research are included. 

Upon the visual inspection of the spectra collected for pure saffron, safflower, and turmeric (Figure 1), we found that the signature of each sample appeared distinct, showcasing different major organic peaks. A closer examination of these spectra revealed low-intensity secondary ions related to minerals, further distinguishing between the three references.

In the low-mass region (below *m*/*z* 100), the positive spectra of these three spices showed signals assigned to potassium ions (K^+^ at *m*/*z* 39) and short hydrocarbon fragments (mainly C_n_H_2n+1_ with n = 2, 3, and 4, and CnH_2n−1_ with n = 3 to 6). Saffron exhibited the highest intensity for the potassium ion, while safflower had the lowest. Concerning the hydrocarbon fragments, safflower showed the highest yield with a broader range of possible combinations, although their relative intensities varied from one replicate to another. Secondary ions with amino groups were predominantly detected in saffron (like C_3_H_8_N^+^ at *m*/*z* 58, C_5_H_3_N^+^ at *m*/*z* 77, and C_4_H_12_N_2_O^+^ at *m*/*z* 104), and to a lesser extent in the turmeric spectrum (C_3_H_8_N^+^). According to Kawecki et al. [19], these peaks are considered a result of only amino acid fragmentation; indeed [19], the first two mentioned peaks are common to most amino acids, while the third one can be attributed to glutamine.

In the mass region between 100 and 600 *m*/*z* considering the saffron spectrum, the most intense peaks detected were fragment ions of both inorganic and organic origins. We attributed the peaks at *m*/*z* 149, 157, 175, and 213 to K_2_PO_2_^+^, K_2_PO_3_^+^, K_2_H_2_PO_3_^+^, and K_3_PO_4_H^+^ ions, respectively. The organic peaks at 184 and 430 remained unassigned. According to the respective mass resolution, the software attributed these ions to undecanoic fatty acid (C_10_H_22_O_2_^+^) and alpha-tocopherol (C_29_H_50_O_2_^+^), respectively, although they have not been reported in the literature for saffron. By contrast, potassium and phosphorus were found to be the predominant minerals in saffron, setting it apart from safflower and turmeric. This is evident in our data where the peaks associated with K^+^ ion and PO_3_^−^ ion and their recombination dominated the saffron spectrum compared to the other two spices. 

In the high-mass region of the saffron positive spectrum (>700 *m*/*z*), multiple clusters of peaks are assigned to crocin, the compound responsible for saffron’s red color. We attributed this pattern of clusters to the fact that crocin includes a family of glucose-esterified crocetin derivatives [21,43,44], which was not the case in the work of Lee et al. [45,46], where crocin was extracted from the gardenia plant and analyzed as a thin layer. The suggested peak assignments with the fragmentation pattern presented in Figure 1b are related to alpha crocin. Although these crocin fragments provided a direct identification of saffron, due to their low intensity during the suggested time of analysis, they were not included in the statistical analysis. 

Regarding the safflower spectrum (Figure 1), a series of cluster ions were found to mainly characterize the mass region above *m*/*z* 100. Considering safflower components, these 14 clusters, spaced by 14 atomic mass units and ranging from *m*/*z* 381 to *m*/*z* 565, were attributed to fragments extracted from the triacylglycerol of a single fatty acid or a combination of two, such as linoleic acid (18:2) with alpha-linolenic acid (18:3) or with palmitic acid (16:0) (refer to Table S1). Indeed, their signature, when analyzed with TOF-SIMS, shows the same pattern, a cluster of five to six ions with a 1 H difference [47,48,49,50,51,52], and their presence in safflower petals ranges between 4% and 8% in total [16,53]. Moreover, the molecular ion of each of these fatty acids was also present in the negative spectrum but at a lower intensity than the fragments of triacylglycerol, which was also present in the negative spectrum between 300 and 600 u.m.a. Certainly, other possibilities should also be considered. The two organic peaks observed at *m*/*z* 113 and 179 with the proposed chemical formulas C_8_H_9_O_3_^+^ and C_9_H_13_O_3_^+^, respectively, can be also assigned as fragmentations of triacylglycerol.

As for the turmeric spectrum (the blue spectrum in Figure 1), the dominant peaks in the mass region above *m*/*z* 120 could be assigned to one of the bioactive compounds in the turmeric sample—curcumin [54,55]. The molecular ion [M+H] with the chemical formula C_21_H_21_O_6_^+^ of curcumin was observed at *m*/*z* 369, accompanied by five proposed fragmentations at *m*/*z* 339, 219, 177, 147, and 137, as illustrated in Figure 1.

### 3.2. Characterization of Saffron Adulterated with Safflower and Turmeric

In Figures S4 and S5, the TOF-SIMS spectra of saffron adulterated with 20% turmeric (Saf80:20Turm) and 20% safflower (Saf80:20Saffl) are shown. Considering a direct comparison of the spectra acquired for pure turmeric (Figure 1) and saffron adulterated with turmeric (Figure S4), it is interesting to note that the peaks associated with the curcumin molecule and its fragmentations in the pure turmeric spectra are absent in the spectra of saffron adulterated with 20% turmeric. Although 20% *w*/*w* is considered a high concentration, the secondary ions emitted from turmeric appear to be masked or suppressed by the saffron matrix, a phenomenon not uncommon in TOF-SIMS analysis [32]. On the other hand, when the spectra of pure safflower and the respective adulterated saffron are compared, the clusters of ions in the mass region between 300 and 600 u.m.a, which are characteristic fingerprints of safflower, persist even in saffron adulterated with safflower, even at lower levels of inclusion. In this context, unraveling the intricacies of SIMS data for the purpose of discriminating between different groups could be more effectively accomplished through the application of multivariate procedures. For major details, refer to the figures in the Supporting Information.

### 3.3. Data Processing and Statistical Analysis

In this study, principal component analysis (PCA) was performed using Spectragui software V2.8, adapted for IONTOF recorded data. Powder samples of pure saffron and saffron adulterated with varying amounts of safflower and turmeric (at 5%, 10%, and 20% *w*/*w* levels for each adulterant) were analyzed repeatedly across different areas and times.

Initially, only the TOF-SIMS spectra obtained from the 20% adulteration level for either turmeric (Saf80:20Turm) or safflower (Saf80:20Saffl) were considered for PCA alongside pure saffron (Saf100). Consequently, three distinct data groups were established and compared. Peaks with a signal-to-background ratio threshold and a minimum count set at 3 and 10,000, respectively, were included in the PCA. This selection aimed to determine whether the peaks with higher yields in their respective spectra, primarily from the organic components of the sample, could capture the differences between the groups.

Before PCA, the selected peaks were normalized to the total spectral intensity and mean-centered. Mean-centering ensured that the observed variance was genuinely due to differences among the samples rather than differences in sample means. The interpretation of the results focused on the first two principal components (PCs) as their combined variances exceeded 98%. As depicted in Figure S6, Saf80:20Saffl (blue group) was well discriminated from Saf80:20Turm (red group) and pure saffron, while no separation was obtained between this last and samples adulterated with turmeric (Saf80:20Turm). A closer examination of the spectra revealed that, for most selected variables, the intra-group variation in their intensities was much higher than the inter-group variation. 

**Figure 2:**
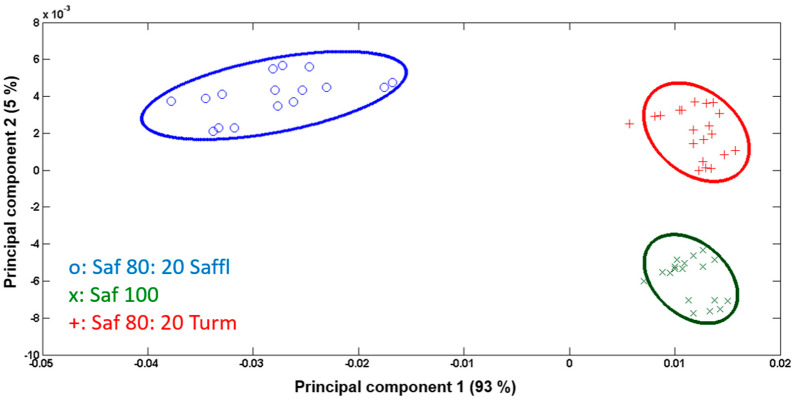
PC1 (93%) vs. PC2 (5%) score plot of pure saffron (green) and saffron adulterated with 20% safflower (blue) and 20% turmeric (red). The colored ellipses around the points define the 95% confidence limit for each sample group.

**Figure 3:**
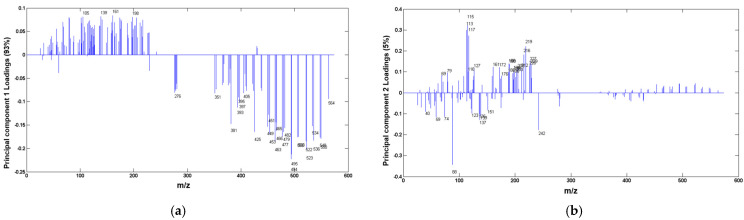
Loading plots of (**a**) PC1 and (**b**) PC2 score plots for TOF-SIMS spectra of pure saffron and saffron adulterated with 20% safflower and 20% turmeric.

To improve the PCA results, the list of peaks for statistical analysis was refined to include only those showing a signal-to-background ratio of 3 but a minimum intensity of 500 counts. Moreover, only the variables with higher variation between the three groups were considered. Consequently, PCA was performed on the base of 146 selected peaks for the three groups, following the same preprocessing steps. The results are depicted in Figure 2 and Figure 3. Only the first two PCs were considered, as their combined variances exceeded 98%.

Figure 2 displays the score plot of the PC1 versus PC2, while Figure 3 illustrates the loadings of each PC versus the corresponding variables. PC1, capturing the highest variation among the samples, distinguishes saffron adulterated with 20% safflower (depicted as blue dots in Figure 2 with negative scores in Figure 3a) from the other two groups. The significant negative loading values for this separation are attributed to the clusters of peaks in the mass region *m*/*z* 300–600.

PC2, which captures a 5% variance, facilitates the statistical separation between saffron pure (green dataset) and saffron adulterated with safflower and turmeric. The variables responsible for this distinction are highlighted in Figure 3b through their negative loadings. Notably, secondary ions at *m*/*z* 88, 136, and 242, which are attributed to C_5_H_8_N^+^, C_8_H_10_NO+, and (C_8_H_11_N)_2_^+^, representing amino acid fragments, play a key role. In fact, saffron is renowned for its elevated protein content compared to safflower and turmeric. Various studies have been conducted to determine the type and quantity of its amino acids [56,57], and the respective profiles were used for differentiating saffron from different geographical origins [57]. Additionally, other variables contributing to the separation of saffron from the other two groups are of mineral origin. Saffron is rich in minerals, with detectable percentages of potassium (~1%), magnesium (0.22%), phosphorus (0.4%), and calcium (0.11%). The identified mineral fragments include Ca^+^, CaOH_3_^+^, K_2_OH^+^, KPOH_2_^+^, and KPO_4_H_3_^+^, with *m*/*z* values of 40, 59, 95, 123, and 137, respectively [20,37,58].

**Figure 4:**
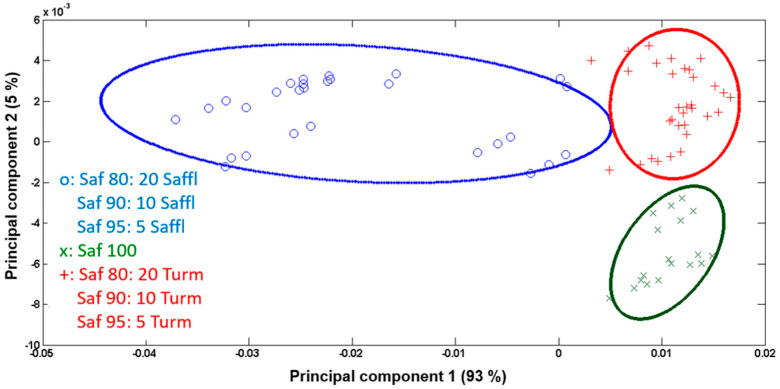
PC1 vs. PC2 score plot of pure saffron (green), saffron adulterated with safflower (blue), and with turmeric (red). The ellipses around the points define the 95% confidence limit for each sample group.

Figure 2 also illustrates the two-dimensional separation among the three groups of samples, analyzed with a 95% confidence limit. PCA successfully discriminated between pure saffron and saffron adulterated at a 20% inclusion level. The intra-group variation is minimal, while the distinction among the three groups is prominently evident. Based on the previous results, PCA was conducted on three new sample groups: pure saffron, saffron adulterated with different percentages of safflower, and saffron adulterated with different percentages of turmeric. The two-dimensional separation of these groups based on the first two components is illustrated in Figure 4. Similar to what was already observed for saffron adulterated with 20% safflower and turmeric, the primary variation among these three groups was attributed to the characteristic peaks of safflower.

The secondary variation, depicted by the loading plot based on PC2, was mostly associated with mineral peaks and amino peaks. While the discrimination between the groups is evident, it is not highly pronounced. In terms of saffron adulterated with safflower at different levels, the intra-group variation was relatively high. As shown in Figure S7, when each level of inclusion is treated as a distinct group, there is a clear separation between these groups and the pure saffron group. The score of Saf95:5Saffl was closer to pure saffron than that of Saf80:20Saffl, as anticipated. Regarding the saffron samples adulterated with turmeric, when each group with a different inclusion level was considered separately, they could not be discriminated from pure saffron.

Authentication is a critical issue in the global trading of saffron. Significant efforts have been made to develop sensitive analytical methods using both targeted and untargeted approaches for the prompt detection of adulterated samples [12]. Various analytical techniques have been developed to discriminate pure saffron from counterfeit products, including chromatographic, spectroscopic, molecular–biological, and biomimetic-based techniques [12]. Recently, mass spectrometry coupled with metabolomics and chemometric tools has been investigated as a reliable method to unveil counterfeit saffron. Rubert et al. proposed an analytical method based on an untargeted metabolic fingerprinting approach using UHPLC–HRMS followed by chemometrics to distinguish saffron origin [59]. They developed both supervised and unsupervised models that successfully distinguished between the protected designation of origin (PDO) saffron and labeled Spanish saffron, revealing fraudulent behavior in more than 50% of samples. Conversely, using a targeted approach, different glycerophospholipids and their lipid oxidation products were identified as significant markers for discriminating PDO Spanish saffron [59]. More recently, Senizza et al. proposed a UHPLC-ESI/QTOF-MS method based on a metabolomic approach followed by multivariate statistical analysis to discriminate between pure saffron and saffron adulterated with other flower parts, as well as to trace its geographical origin [60]. By focusing on the phenolic fraction, they used both unsupervised hierarchical clustering and supervised orthogonal partial least-square discriminant analysis to achieve good separation between authentic saffron and saffron adulterated with other floral components, even at an inclusion level of 5%. They identified different anthocyanins, glycosidic flavonols, flavonoid molecules, and other specific compounds as validated markers for style and origin adulteration [60]. Additional targeted MS-based methods exploiting specific marker compounds for saffron authentication were described by Guijarro-Díez et al. and Aiello et al. [61,62].

Despite their promise, these methods are limited by the complexity of sample preparation and the time required for extraction and chromatographic runs. To expedite saffron authentication, our team recently demonstrated the discriminatory potential of metabolic profiles obtained using optimized DART-HRMS conditions, with an unsupervised multivariate analysis based on hierarchical cluster analysis and principal component analysis to rapidly identify saffron adulterated with safflower or turmeric, detecting inclusion levels as low as 5% [22]. In this study, we investigated and optimized a method based on the TOF-SIMS technique for rapid saffron authentication, using the same set of samples. This approach presents notable advantages, including the elimination of sample extraction or preparation steps and the requirement of only a few milligrams of sample material, thus enhancing cost-effectiveness. As discussed earlier, our findings revealed that using an unsupervised list for PCA statistical analysis enabled effective discrimination between pure saffron and saffron adulterated with safflower at inclusion levels below 20%. Additionally, using a supervised list for PCA facilitated discrimination between pure saffron and saffron adulterated with safflower and turmeric, also at inclusion levels below 20%, due to distinct markers from saffron and safflower. In both scenarios, we observed robust grouping and differentiation when comparing pure saffron with safflower-adulterated samples across the varied inclusion levels examined.

### 3.4. Semi-Quantification of Safflower

It is known that 99% of escaped ions from the sample exist in a neutral state, and the probability of ionization or de-excitation as these ions pass through the surface is significantly influenced by the electronic properties of the matrix, the so-called “matrix effect” [32]. Therefore, fragmentation under the beam and the reorganization on the surface of the secondary ions primarily depend on the sample’s constituents and their quantities. Therefore, the emission yield (peak intensity) of secondary ions could be influenced by the matrix composition, sometimes leading to either enhanced or suppressed emission of specific fragments. Consequently, TOF-SIMS is not considered a direct quantitative technique. However, in some cases, it can allow for relative concentration analysis when comparing samples with different concentrations of a given compound within the same matrix.

**Figure 5:**
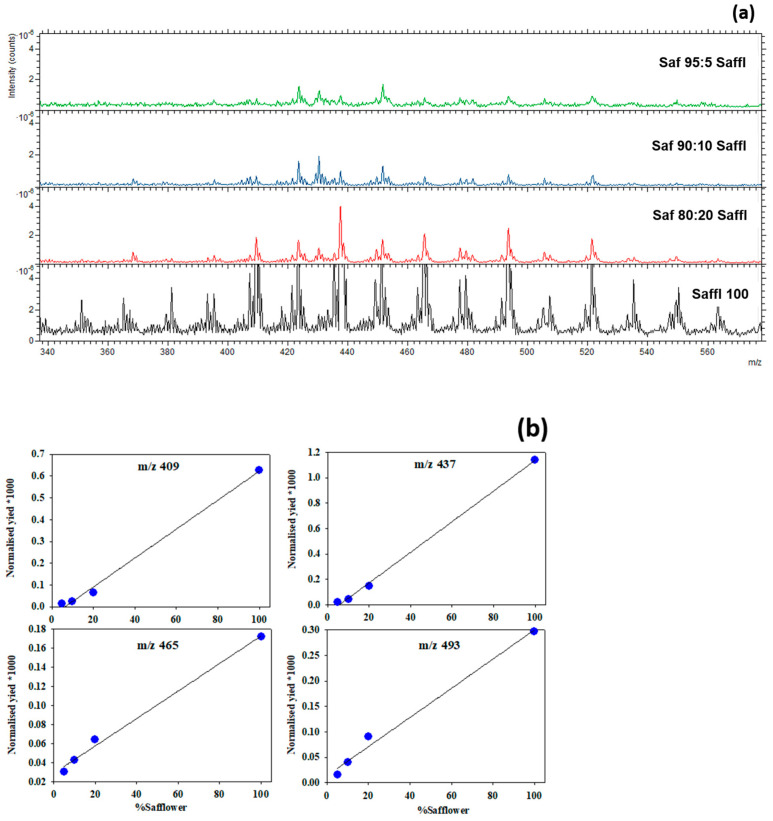
Variation in the characteristic peaks of safflower in the adulterated saffron samples at 5% (Saf 95:5 Saffl), 10%(Saf 90:10 Saffl), and 20%(Saf 80:20 Saffl) and in pure safflower (Saffl 100) presented (**a**) by overlaying their normalized mass spectra and (**b**) comparing the variation in the 4 major peaks’ intensities with safflower % in the samples.

In this part of the study, we focused on the emission yield of the characteristic peaks of safflower, which was found to be in the mass range between 300 and 600. These peaks, showing higher loading in PC1, contribute to the significant variation observed between adulterated samples with safflower and other samples. Figure 5 illustrates the variations in the normalized emission yield of the characteristic peaks of saffron with the percentage of inclusion. A proportionality between peak intensities and safflower percentages is well defined, especially in the percentage range below 20%. The variation in the normalized intensities of the presented peaks (*m*/*z* 409, 437, 465, and 493) follows a linear trend, with the percentage of safflower achieving an R^2^ value over 0.98 for the fitted curve. 

Each point represents an average of the analyzed regions of each sample, with the standard deviation within the size of the points. Even when excluding the data points of pure safflower peaks from the calculation, given its distinct matrix from the adulterated saffron with safflower, the results still indicate a linear variation with an R^2^ of the same magnitude. These findings are promising considering the limitations of the current techniques in performing quantification analysis. The results obtained in this work have a dual scope. Firstly, they demonstrate that the developed method is suited for the rapid screening and qualitative assessment of a saffron sample under study: As has been shown, the presence of specific peaks provides clear evidence of safflower inclusion. Secondly, for the assessment of the percentage of inclusion, the calculation of the normalized intensities of these peaks should suffice.

## 4. Conclusions

A reliable method was successfully developed to verify saffron authenticity by discriminating pure saffron from saffron adulterated with turmeric and safflower at inclusion levels below 20%. This was achieved by analyzing a few milligrams of powder samples without any prior treatment using the TOF-SIMS technique. The spectra of pure saffron, pure turmeric, and pure safflower were recorded for the first time. Despite the complexity of peak assignment, some bioactive compounds, fatty acids, amino acids, and mineral fragments were identified. A supervised list of peaks was considered for PCA. The discrimination between the groups of pure saffron and adulterated saffron with turmeric and with safflower, separately, at 5%, 10%, and 20% inclusion levels, was evident in a new bi-dimensional representation, although it was less pronounced when only the 20% adulteration level was considered. The variation between the groups is attributed to the characteristic peaks of safflower and the amino group peaks and mineral peaks of saffron. For saffron adulterated with turmeric, the different levels of inclusion were not easily distinguishable from pure saffron when considered individually. In the case of saffron adulterated with safflower, the level of inclusion can also be calculated from the normalized safflower characteristic peak intensities, which exhibit a linear variation with the percentage of safflower in the saffron matrix.

## Data Availability

The original contributions presented in the study are included in the article/Supplementary Material, further inquiries can be directed to the corresponding authors.

## Supplementary Materials

The following supporting information can be downloaded at: https://www.mdpi.com/article/10.3390/foods13132033/s1, Figure S1: Positive and negative spectra of pure saffron powder analyzed with 0.1 pa Bi3+ current and a raster size of 500 × 500 µm^2^; Figure S2: Positive and negative spectra of pure safflower powder analyzed with 0.1 pa Bi3+ current and a raster size of 500 × 500 µm^2^; Figure S3: Positive and negative spectra of pure turmeric powder analyzed with 0.1 pa Bi3+ current and a raster size of 500 × 500 µm^2^; Table S1: Proximate and photochemical composition of saffron, safflower, and turmeric powders as reported in the literature and chemical structure of some compounds; Figure S4: Positive spectra overlay of pure turmeric and saffron adulterated with 20% turmeric (inverted). The asterisk (*) symbol denotes the signature peaks of curcumin molecule; Figure S5: Positive spectra overlay of pure safflower and saffron adulterated with 20% safflower (inverted). The asterisk (*) symbol denotes the signature peaks of turmeric; Figure S6: PC1 vs. PC2 score plot of pure saffron (green), saffron adulterated with 20% of safflower (Saff80:20Safl, blue) and turmeric (Saff80:20Turm, red). The ellipses around the points define the 95% confidence limit for each sample group; Figure S7: PC1 vs. PC2 score plot of pure saffron (Saf) and the three different groups of saffron adulterated with 5%, 10%, and 20% of safflower (saffl) and the two groups of saffron adulterated with 5 and 20% of turmeric (Turm). The ellipses around the points define the 95% confidence limit for each sample group.
