# Supplementary material for: Time-of-Flight Secondary Ion Mass Spectrometry Coupled with Unsupervised Methods for Advanced Saffron Authenticity Screening
Source: Foods. 2024 Jun 27;13(13):2033. doi: 10.3390/foods13132033 (PMC11241374; doi:10.3390/foods13132033)
Supplement: Supplementary file 1 [file foods-13-02033-s001.zip › foods-3027648-supplementary.pdf]

Supplementary Materials

Time of Flight Secondary Ion Mass Spectrometry Coupled with Unsupervised Methods for Advanced Saffron Authenticity Screening

Elisabetta De Angelis[1], Omar Al-Ayoubi[2], Rosa Pilolli[1], Linda Monaci[1*] and Alice Bejjani[2*]

1 Institute of Science of Food Production, National Research Council of Italy, Via G. Amendola 126/O, 70126 Bari, Italy

2 Lebanese Atomic Energy Commission, National Council for Scientific Research, Riad El Solh 107 2260 Beirut, Lebanon

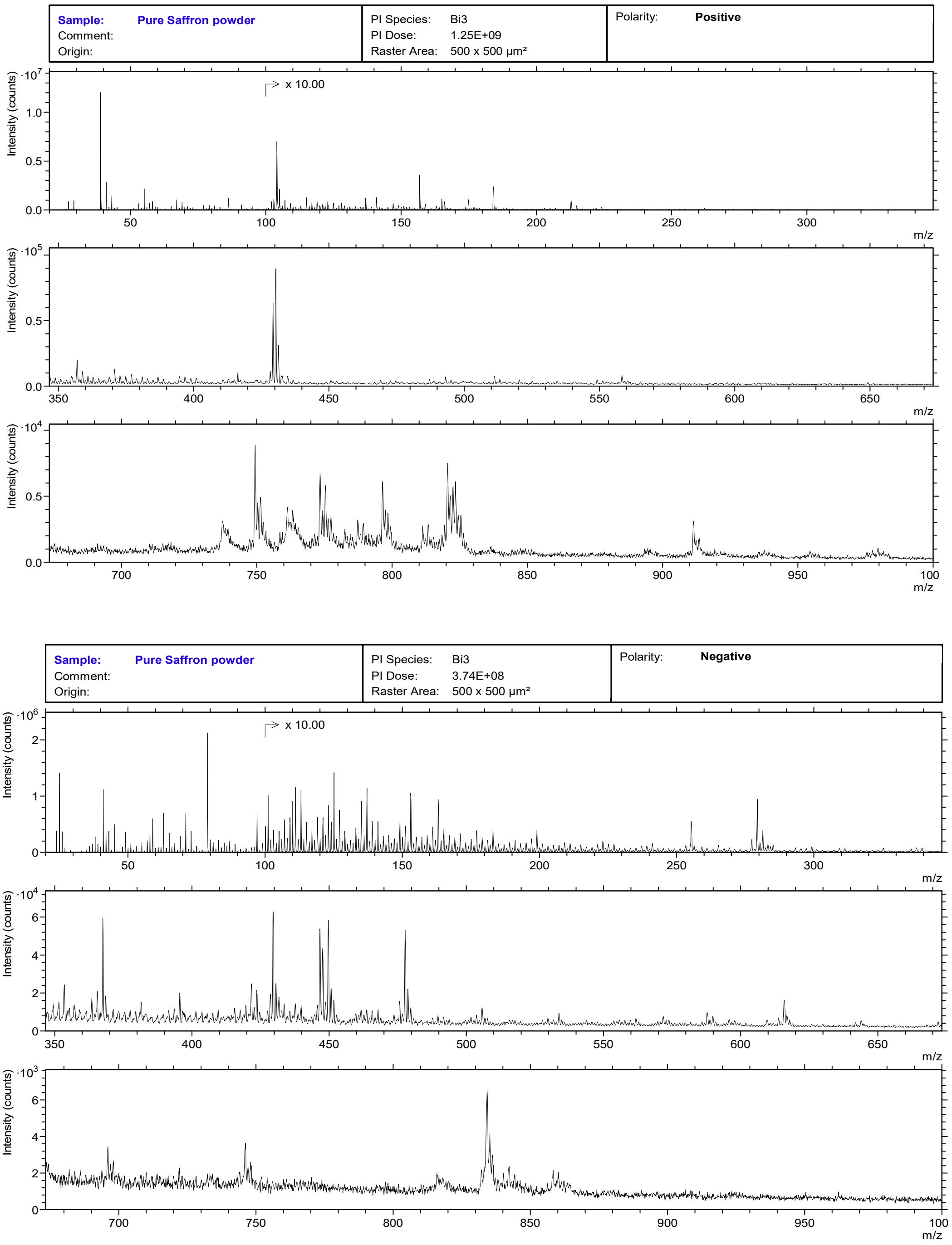

Figure S1. *Positive and negative spectra of pure saffron powder analyzed with 0.1 pa* Bi_3^+ *current and a raster size of 500×500 µm*^{2}.

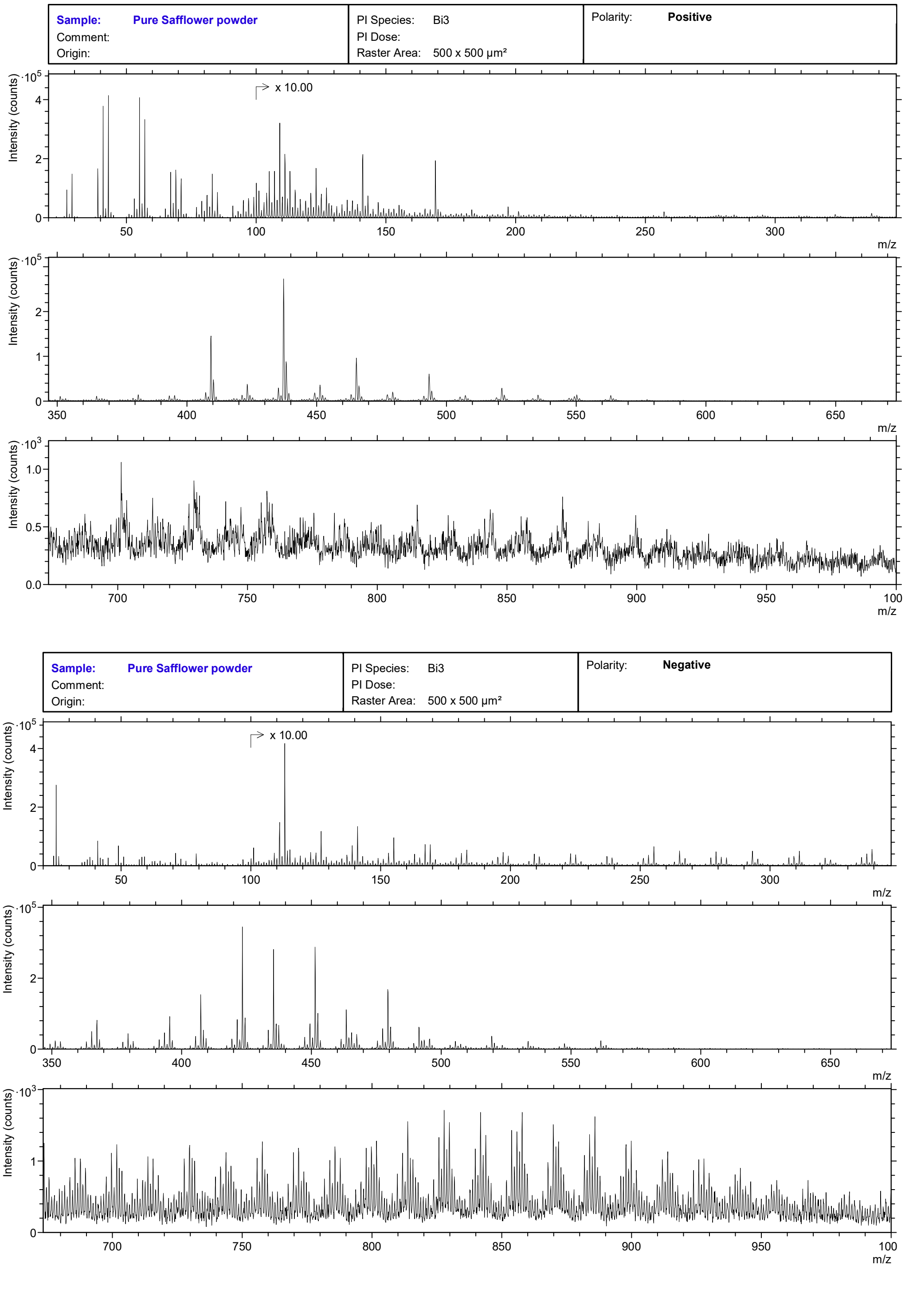

Figure S2. *Positive and negative spectra of pure safflower powder analyzed with 0.1 pa* Bi_3^+ *current and a raster size of 500×500 μm*^{2}

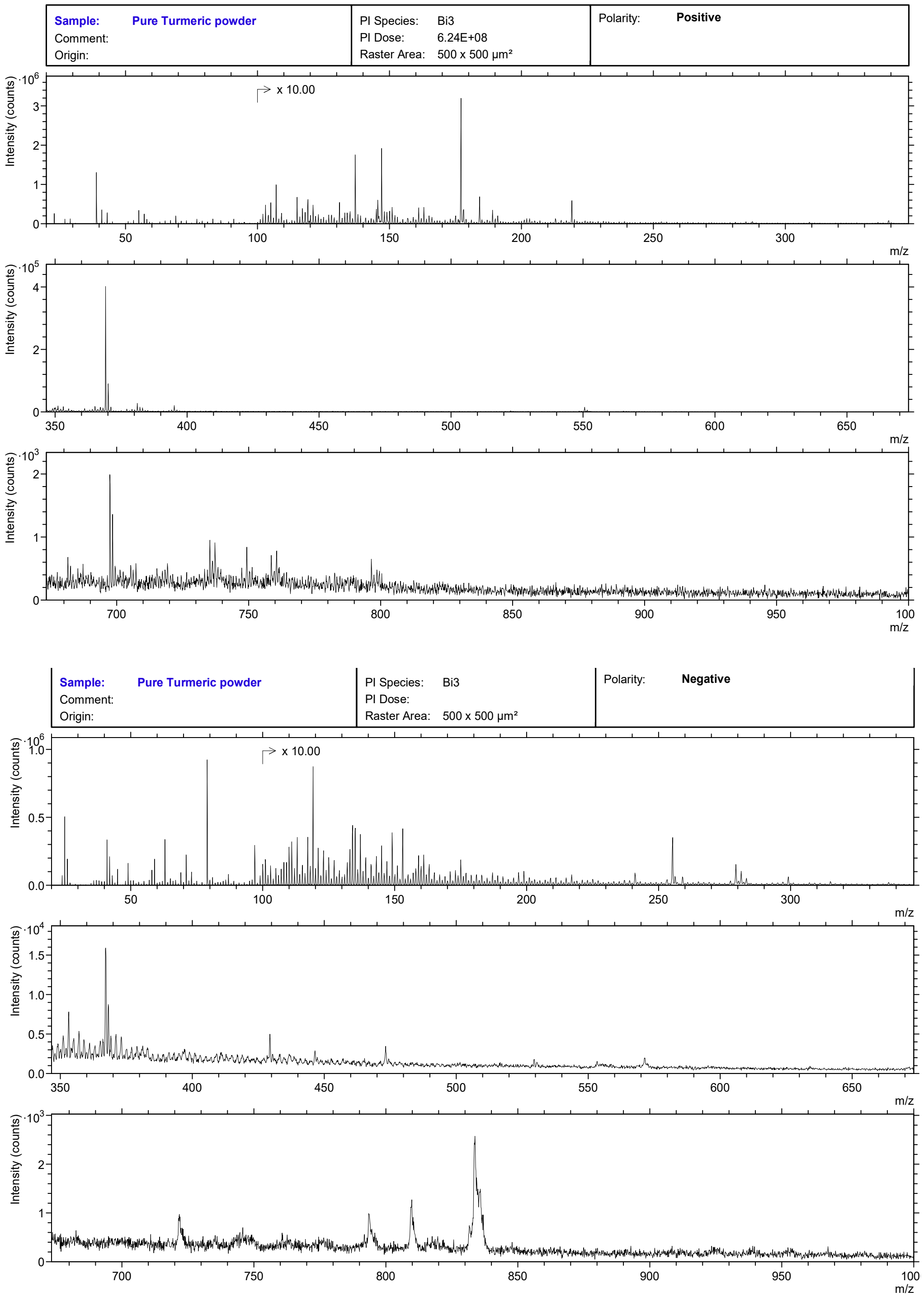

Figure S3. *Positive and negative spectra of pure turmeric powder analyzed with 0.1 pa* Bi_3^+ *current and a raster size of 500×500* μm^2

Table S1. *Proximate and photochemical composition of saffron, safflower and turmeric powders as reported in the literature and chemical structure of some compounds.*

**Compound classification**	**Content (in mg/g or in %)**	**Chemical structure (with some proposed fragments)**
**Saffron (stigma)**		
Carotenoids (mostly crocin)	6-16%	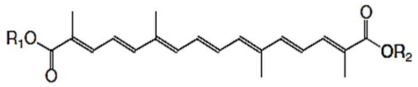 Crocetin with R1 = R2 = Gentiobiosyl or Glucosyl sugar moiety 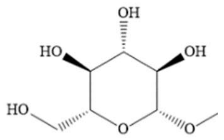 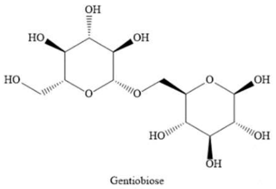 Related peaks with m/z 771, 758, 798 and 820
Terpenoid (mostly picrocrocin: the glucoside precursor of safranal)	1-13%	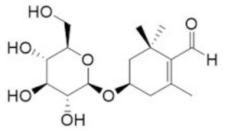
protein	12-14%	Related peaks with m/z 58, 77 and 104
pectin	6-7%	
Dextrin	9-10%	
Flavonoids (mostly reported anthocyanin and keampferol)	~ 9.68 and 12.6 mg/g respectively	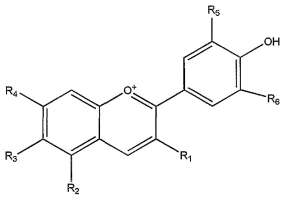 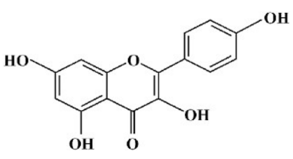

Minerals	K 8.2 → 12.2 mg/g<br>Mg 1.1 → 1.76 mg/g<br>P 3.2 → 4.4 mg/g	Related peaks with m/z 24, 39, 95, 141, 157, 175
**Safflower (petals)**		
Quinochalone<br>(Mostly Carthamin)	0.83%	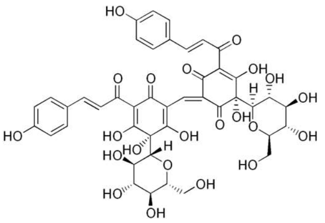
Flavonoid (kaempferol)	High content	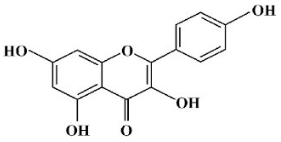
protein	1.8%	
Fatty acid<br>(Mostly linoleic acid (18:2), alpha linolenic acid (18:3) and palmitic acid (16:0))	4-8%	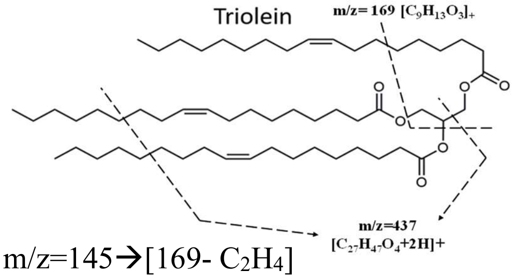<br>m/z=145→[169- C_2H_4]<br>m/z=409→ [437- C_2H_4]<br>m/z=465→[437+ C_2H_4]<br>m/z=493→ [437+ $(C_2H_4)_2$]<br>m/z=521→[437+$(C_2H_4)_3$]
Crude fibers	11.6%	
ash	10.8%	
Minerals	Ca 0.53 mg/g<br>Mg 0.287 mg/g<br>Fe 0.0073mg/g	
stigmasterol	Reported as high content	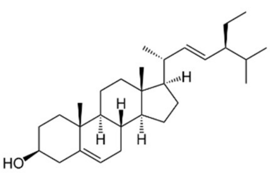

Beta-daucosterol	Reported as high content	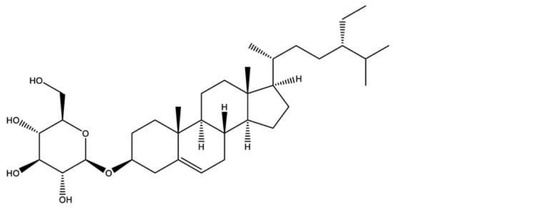
**Turmeric (rhizome)**		
Curcuminoids (Mostly curcumin)	2-15%	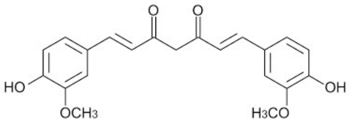<br>Related peaks with m/z 137, 147, 177, 219, 339 and 369
flavonoids	~0.4 %	
Essential oil	3-7%	
Carbohydrates	~63%	
water	~13%	

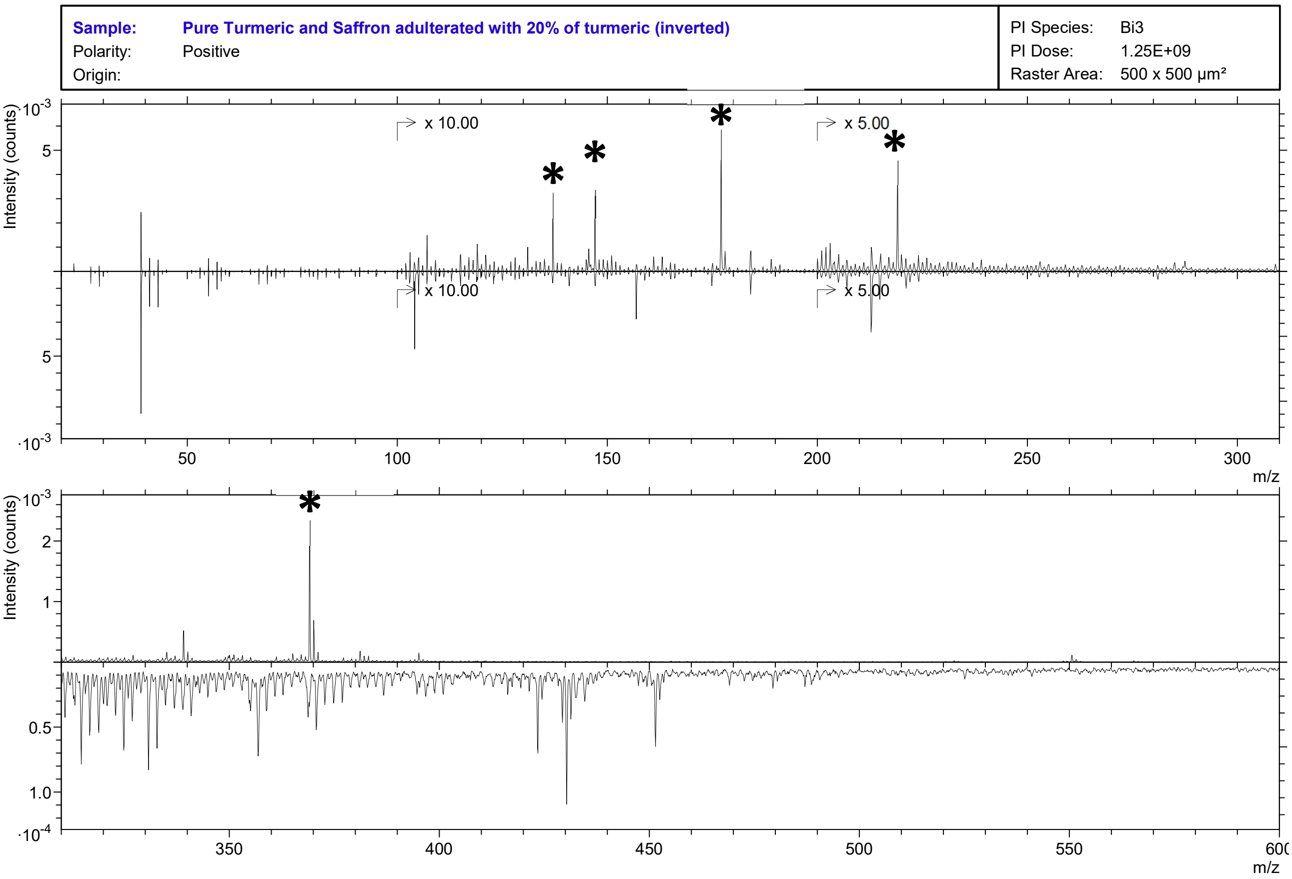

Figure S4. *Positive spectra overlay of pure turmeric and saffron adulterated with 20% turmeric (inverted). The asterisk (*) symbol denotes the signature peaks of curcumin molecule.*

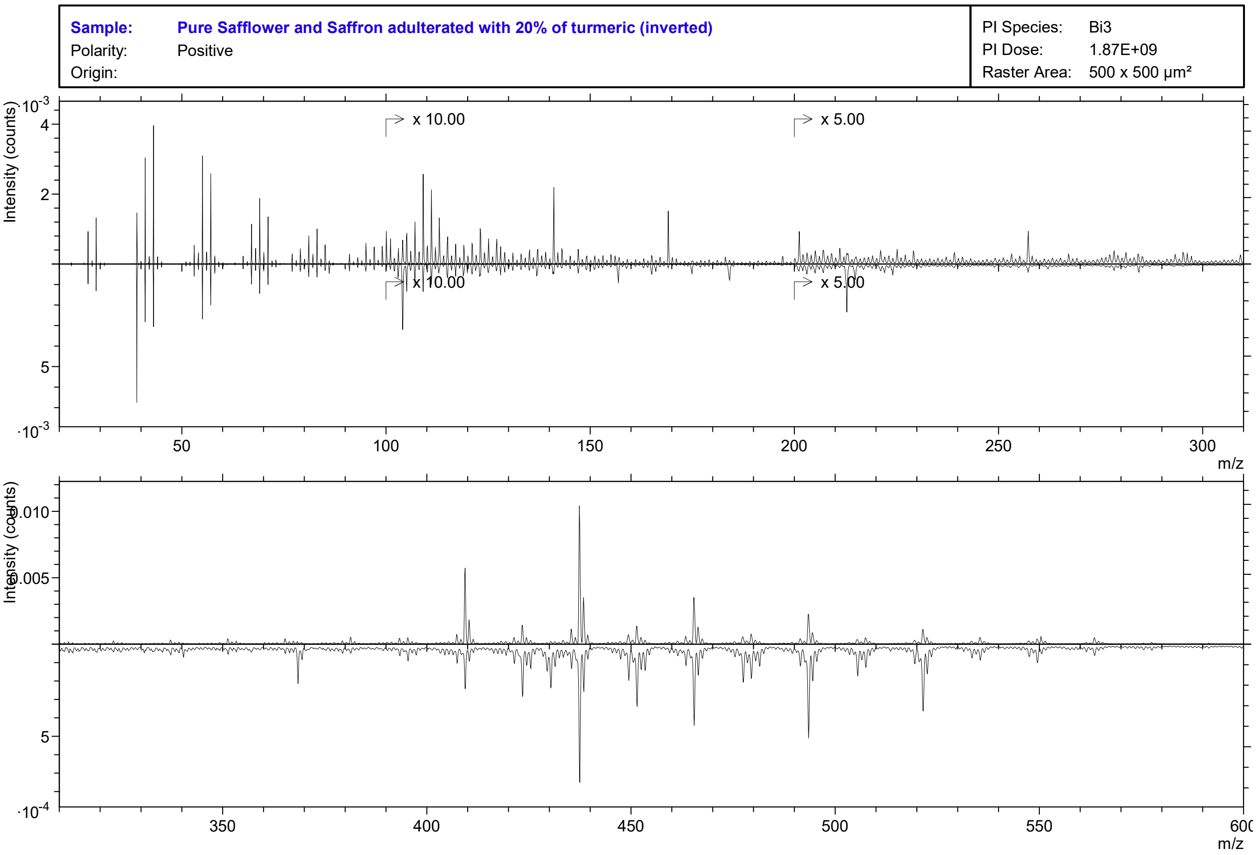

Figure S5. *Positive spectra overlay of pure safflower and saffron adulterated with 20% safflower (inverted). The asterisk (*) symbol denotes the signature peaks of turmeric.*

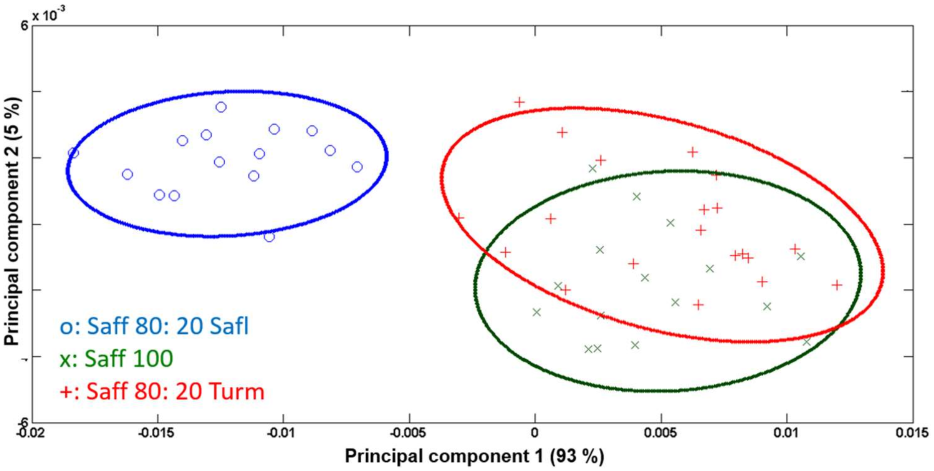

Figure S6. *PC1 vs. PC2 scores plot of pure saffron (green), saffron adulterated with 20% of safflower (Saff80:20Safl, blue), and with turmeric (Saff80:20Turm, red). The ellipses around the points define the 95% confidence limit for each sample group*

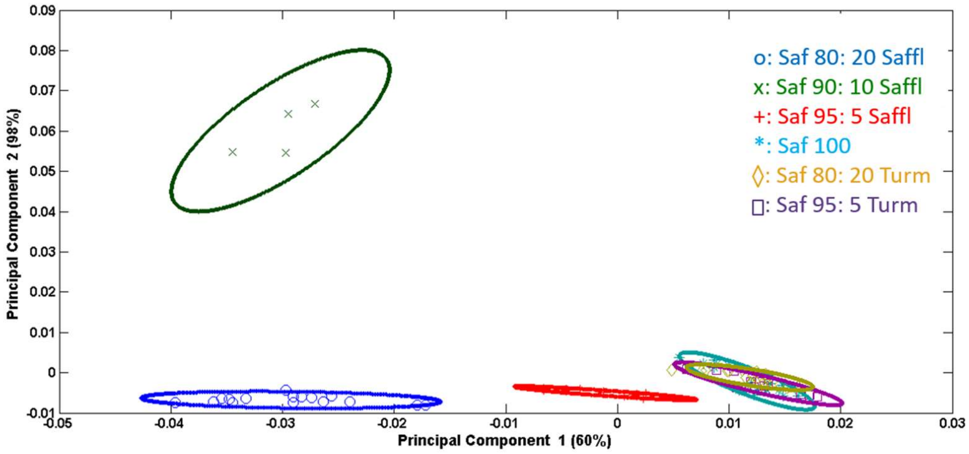

Figure S7. *PC1 vs. PC2 scores plot of pure saffron (Saf) and the three different groups of Saffron adulterated with 5%, 10% and 20% of Safflower (saffl)and the two groups of saffron adulterated with 5 and 20% of turmeric (Turm). The ellipses around the points define the 95% confidence limit for each sample group*
